# The prevalence of ESBL-microorganisms among students of Southeast Asia living in Kazakhstan

**DOI:** 10.1186/1753-6561-9-S7-A28

**Published:** 2015-10-27

**Authors:** Svetlana Kolesnichenko

**Affiliations:** 1 Faculty of General Medicine, Karganda State Medical University, Karganada, Kazakhstan

## Background

There are more and more information about ESBL-producing organisms wide spreading in Europe and Southeast Asia in recent years [1]. A large amount of students from Southeast Asia are staying in Kazakhstan, which raises the question about the distribution of microorganisms and aggravation of epidemiological situation. In our study we investigated the prevalence of ESBL-producing microorganisms among Southeast Asian students studying in Kazakhstan.

## Methods

Material for the study – feces. 139 fecal samples from foreign students from India and Pakistan were investigated in 2012 and 291 samples in 2013. The samples were examined for the presence of ESBL-producing in a medium containing ceftriaxone (6 mkg/ml). Identification of isolated microorganisms was performed by using MALDI-TOF spectrometer Microflex and software complex Biotyper of Bruker Daltoniks. Confirmation of ESBL-production was performed by double-discs method. Sensitivity to antibiotics was determined by disk diffusion method.

## Results

In 2012, as a result of screening plates with ceftriaxone from 139 stool samples 48 strains were cultivated. Those are 35 (72.9%) *Escherichia coli* strains and 1 (2%) strain of *Klebsiella pneumonia*. After determining the sensitivity of these strains to antibiotics by disk diffusion method, ESBL-producing confirmed in 24 samples: 23 (47.9%) ESBL-positive *E. coli* strain and 1 (2%) strain of *K. pneumonia*. According to the results of study in 2013, 280 microorganisms were cultivated from 292 stool samples, including 88.2% of *E. coli* (95% CI 20.24-34.14). Other types of microorganisms were encountered in rare cases. 86% *E. coli* (240 strains) were ESBL-producers. Significant differences in the two methods: the method of double-discs and agar plates containing ceftriaxone was not obtained (p<0.05).

## Conclusions

The survey for 2012 was obtained 49.9% ESBL-producing strains, for 2013 - 86%, which means an increase in the prevalence of ESBL-producing strains. Using the agar plates containing ceftriaxone for ESBL screening significantly reduces the time and increases the level of studies of antibiotic-resistant strains of isolation.
